# Efficacy and exploratory analysis of potential mechanisms of stellate ganglion block in alleviating sleep disturbance in generalized anxiety disorder: a randomized controlled trial excluding comorbid depression

**DOI:** 10.3389/fneur.2025.1554841

**Published:** 2025-05-07

**Authors:** Na Liu, Qinying Ma, Moqing Zhou, Lin Yang, Wenyuan Wang, Yanyong Wang

**Affiliations:** Department of Neurology, The First Hospital of Hebei Medical University, Shijiazhuang, Hebei, China

**Keywords:** stellate ganglion block, generalized anxiety disorder, sleep disturbance, efficacy, mechanism

## Abstract

**Objective:**

To investigate the efficacy and mechanisms of stellate ganglion block (SGB) in treating generalized anxiety disorder (GAD) with sleep disturbance, excluding patients with comorbid depression.

**Methods:**

This double-blind randomized controlled trial (RCT) enrolled 128 patients with GAD (Hamilton Anxiety Scale [HAMA] > 14, Generalized Anxiety Disorder 7-item Scale [GAD-7] ≥ 5) and sleep disturbance (Pittsburgh Sleep Quality Index [PSQI] ≥ 15), randomized to receive SGB (*n* = 64, 4 ultrasound-guided 1% lidocaine injections) or conventional treatment (*n* = 64, cognitive behavioral therapy [CBT] + estazolam 1–2 mg/day). Outcomes included anxiety (HAMA), depression (Hamilton Depression Scale [HAMD]), sleep quality (PSQI), polysomnography (PSG), and neurotransmitter levels (norepinephrine [NE], serotonin [5-HT], neuropeptide Y [NPY]).

**Results:**

After 4 weeks, SGB demonstrated higher efficacy (98.4% vs. 89.1%, *p* = 0.028) and greater reductions in HAMA (9.36 ± 2.34 vs. 11.87 ± 2.71, *p* < 0.001) and HAMD scores (6.87 ± 2.01 vs. 8.09 ± 2.04, *p* < 0.001). PSQI improved significantly in the SGB group (5.74 ± 1.64 vs. 8.03 ± 1.86, *p* < 0.001), with increased total sleep time (TST) (429.76 ± 33.22 vs. 391.13 ± 30.76 min, *p* < 0.001) and efficiency (90.23 ± 13.29% vs. 86.34 ± 12.84%, *p* < 0.001). Neurotransmitter analysis showed reduced NE (289.43 ± 51.68 vs. 253.78 ± 57.12 pg./mL, *p* < 0.05) and increased 5-HT (138.56 ± 19.73 vs. 124.93 ± 18.44 ng/mL, *p* < 0.05) and NPY (453.21 ± 73.41 vs. 402.34 ± 68.12 pg./mL, *p* < 0.05). Adverse events were comparable (6.25% vs. 3.13%, *p* = 0.403).

**Conclusion:**

SGB effectively improves GAD symptoms and sleep quality in patients without comorbid depression, potentially via modulation of NE, 5-HT, and NPY pathways. The exclusion of psychiatric comorbidities enhances the specificity of these findings.

## 1 Introduction

GAD is a common mental health disorder characterized by excessive and persistent worry, accompanied by symptoms of autonomic dysfunction and hypervigilance. It is one of the most prevalent psychiatric disorders in clinical practice (1). The primary features of GAD include uncontrollable fear, anxiety, and persistent worrying, often accompanied by somatic, behavioral, and cognitive disturbances, as well as sleep disorders. These symptoms significantly impair the physical and mental well-being of affected individuals.

Epidemiological studies suggest that the global prevalence of GAD is approximately 7.3%, and the rapid socioeconomic development, high-intensity work pressures, and environmental stressors in modern society are among the key factors contributing to the onset of this disorder (2). Sleep disturbances are one of the most common comorbidities of GAD. Prolonged sleep deprivation can lead to endocrine imbalances, immune system dysfunction, and exacerbate depressive symptoms, further worsening the overall condition of GAD (3). Among the most common symptoms of GAD with sleep disturbances are difficulties falling asleep and persistent anxiety about sleep. This creates a vicious cycle where anxiety exacerbates sleep problems, and poor sleep in turn increases anxiety, severely impacting the patient’s health, daily life, and work productivity (4, 5).

Current clinical treatments for GAD with sleep disturbances predominantly involve medications such as benzodiazepines and selective serotonin reuptake inhibitors (SSRIs) (6–8). These treatments work by suppressing central nervous system activity to induce passive sleep. However, long-term use of such drugs can lead to physical and psychological dependence and is often associated with a range of side effects (9).

SGB, a technique that targets the cervical sympathetic ganglia, has been used in the management of conditions such as post-mastectomy syndrome, regional pain syndrome, and post-traumatic stress disorder (PTSD). Recent studies have shown that SGB can alleviate anxiety symptoms, reduce sympathetic nervous system hyperactivity, and improve sleep quality in these patients (9, 10). Furthermore, it has been suggested that SGB may regulate the autonomic nervous system and circadian rhythms, offering a potential therapeutic approach for GAD with sleep disturbance (11, 12). However, research on the use of SGB in treating GAD with sleep disturbances is still limited, and the mechanisms underlying its effects remain poorly understood.

Therefore, this study aims to explore the efficacy of SGB in treating GAD with sleep disturbances and investigate the potential mechanisms through which this intervention exerts its therapeutic effects. By assessing clinical outcomes, sleep parameters, neurotransmitter levels, and associated adverse events, this research seeks to provide a better understanding of the role of SGB in managing GAD and sleep disorders.

## 2 Materials and methods

### 2.1 Participant enrollment workflow

A total of 143 potential participants were initially identified through electronic health record (EHR) screening using ICD-10 codes for GAD and PSQI scores ≥15. During preliminary screening, 15 patients were excluded: 4 due to severe comorbidities (coronary artery disease, hyperthyroidism), 3 for active substance abuse, 3 for concurrent psychiatric disorders (schizophrenia/depression), and 5 who declined preliminary eligibility assessment. The remaining 128 patients underwent structured diagnostic interviews conducted independently by two board-certified psychiatrists to confirm GAD diagnosis. Following baseline eligibility verification, all 128 patients were randomized using a computer-generated block sequence (block size = 4, SPSS v26.0) with allocation concealment maintained through sequentially numbered, sealed opaque envelopes. Throughout the intervention phase, zero dropouts occurred due to implemented retention strategies including weekly medication diary monitoring, subsidized transportation for clinic visits, and 24/7 telehealth support for adverse event reporting. Consequently, 128 patients (64 per group) completed the trial with fully analyzable datasets, maintaining the integrity of the original randomization scheme (see Supplementary Figure 1 for full enrollment details). Demographic characteristics (age, gender, baseline HAMA/PSQI scores) of the declining patients showed no significant differences compared to enrolled participants (*p* > 0.05), minimizing potential selection bias. The study protocol complies with the relevant requirements of the Declaration of Helsinki as issued by the World Medical Association. This study was approved by the Ethics Committee of The First Hospital of Hebei Medical University (No. 2019-S00212).

### 2.2 Inclusion and exclusion criteria

Inclusion criteria:

1. Diagnosis of GAD according to the *International Classification of Mental and Behavioral Disorders, 10th Edition (ICD-10)* (13), with a Hamilton Anxiety Scale (HAMA) (14) score >14 and GAD-7 score ≥5 (15);
2. PSQI (16) score ≥15;
3. Age 20–69 years;
4. Patients must be conscious, capable of communication, and able to understand instructions;
5. Signed informed consent.

Exclusion criteria:

1. History of alcohol or substance abuse within the past year;
2. Severe physical comorbidities (e.g., coronary artery disease, arrhythmia, chronic obstructive pulmonary disease, bronchial asthma, hyperthyroidism, brain tumors);
3. Withdrawal from treatment for any reason;
4. Active infectious diseases;
5. Hepatic or renal dysfunction;
6. Known allergies to anesthetic agents;
7. History of neurosurgical procedures or planned neurosurgery;
8. Long-term use of opioids or sedative-hypnotic medications;
9. Comorbid psychiatric disorders (e.g., schizophrenia, depression);
10. History of neurological disorders;
11. Hearing or visual impairments interfering with scale assessments;
12. Endocrine disorders (e.g., pituitary gland diseases).

### 2.3 Sample size calculation

The sample size was calculated using the formula:

$n=2{\left[\left(Z_{1-\alpha }+Z_{1-\beta }\right)s/\sigma \right]}^{2}$

where *α* = 0.05, *β* = 0.01, and the initial calculated sample size was *n* = 109. In accordance with regulations from the China Food and Drug Administration (CFDA), a 15% dropout rate was considered. The final adjusted sample size was determined as:

$n=108\times 1/\left(1–0.15\right)=128.12\approx 128$

Thus, this study enrolled 128 patients with GAD comorbid with sleep disturbances as research subjects.

### 2.4 Blinding method

This study adhered to a double-blind design. Throughout the trial, patients, their families, surgeons, nurses, laboratory technicians, and data analysts were blinded to group assignments. All parties involved strictly maintained independence and avoided communication regarding treatment allocation to ensure unbiased implementation and evaluation. Blinding was achieved through the following measures:

- Anesthetic agents and placebo solutions were prepared in identical syringes by an independent pharmacist.
- Treatment codes were sealed in opaque envelopes and only unblinded after final data analysis.

### 2.5 Treatment methods

#### 2.5.1 Control group

The control group received psychological intervention combined with estazolam tablets. To ensure consistency across participants, all psychological interventions were standardized and delivered by clinicians who underwent comprehensive training in CBT theory and practice for GAD and sleep disturbances. A strict protocol was followed, with regular internal reviews and supervision. The psychological intervention consisted of standardized CBT with the following components:

- Disease education: patients received simplified explanations about the etiology of GAD comorbid with sleep disturbances, the therapeutic mechanisms of SGB, and prognosis. This aimed to help patients develop an accurate understanding of the disorder, correct misconceptions, and reduce anxiety and fear stemming from disease-related uncertainty.
- Cognitive restructuring: patients were guided to identify and challenge negative thought patterns. Therapists emphasized positive aspects of treatment progress and encouraged patients to adopt rational, adaptive thinking to replace mal catastrophic cognitions.
- Behavioral experiments: patients were gradually exposed to feared triggers to test the validity of their negative predictions through real-world experiences. This evidence-based approach aimed to modify irrational beliefs and reduce avoidance behaviors.
- Relaxation training: deep breathing, progressive muscle relaxation, and mindfulness meditation. These interventions targeted physiological arousal (e.g., muscle tension) and emotional distress, thereby alleviating anxiety and improving sleep quality.
- Sleep hygiene education: patients were instructed to adopt healthy sleep practices, such as maintaining consistent sleep–wake schedules, optimizing sleep environments (e.g., quiet, comfortable settings), and avoiding electronic device use before bedtime. These strategies aimed to regulate circadian rhythms and enhance sleep efficiency (SE).

Intervention protocol: each session lasted 45 min, conducted twice weekly over 4 consecutive weeks (total 8 sessions per patient).

Medication: estazolam tablets (manufactured by Beijing Yimin Pharmaceutical Co., Ltd., approval number: National Drug Standard H11020898) were administered at a dose of 1 mg once daily before bedtime. For patients showing inadequate response, the dosage was adjusted up to 2 mg/day under close monitoring. During the treatment period, all patients did not use any medications that could affect hormone levels in the body. The duration of the treatment was 4 weeks.

#### 2.5.2 Study group

On the basis of the intervention in the control group, patients in the study group underwent SGB treatment. The procedure was performed with the patient in a supine position, with the neck slightly extended. After routine disinfection of the neck skin, the ultrasound probe was placed at the C6 level, and the target areas were the esophagus, trachea, thyroid gland, internal jugular vein, carotid artery, prevertebral fascia, and longus colli muscle. For the left-side ultrasound-guided stellate ganglion block (U-SGB), the ultrasound probe was positioned between the trachea and carotid artery, and slight pressure was applied to ensure lateral deviation of the carotid artery. The probe was moved closer to the longus colli muscle. Under ultrasound guidance, the puncture needle was inserted laterally through the prevertebral fascia into the longus colli muscle, without directly entering the muscle. The needle was inserted using a plane technique until the target site was reached. After confirming no blood return during the backflow, inject 5 mL of 1% lidocaine solution, alternating sides, once a day for a total of 4 consecutive times. The entire procedure was monitored using ultrasound to ensure proper diffusion of the drug and injection technique. Vital signs were closely monitored throughout the procedure. Following successful blockage, Horner’s syndrome was observed, including miosis, ptosis, enophthalmosis, conjunctival congestion, increased skin temperature in the face, neck, and palms, and cessation of sweating. Patients were advised to avoid alcohol, smoking, ensure a regular sleep schedule, and avoid spicy foods during treatment.

### 2.6 Observation indicators

#### 2.6.1 Primary outcomes

##### 2.6.1.1 Clinical efficacy

One month after treatment, clinical efficacy was evaluated using the HAMA (14) and Sleep Disturbance Rating Scale (SDRS) (16).

Evaluation criteria:

1. Significant improvement: anxiety and insomnia symptoms were almost completely resolved, and sleep was restored to normal. The HAMA and SDRS scores returned to normal, with continuous sleep ≥8 h at night and increased daytime energy.
2. Effective: anxiety and insomnia symptoms were significantly alleviated, and mental and sleep states returned to near normal. HAMA and SDRS scores showed an improvement of ≥50%, with continuous sleep of 3–8 h at night and moderately increased daytime energy.
3. Ineffective: no improvement in anxiety or insomnia symptoms, with persistent mental and sleep disturbances. HAMA and SDRS scores showed improvement of <50%, continuous sleep time unchanged, and poor daytime energy, or the condition worsened.

$\begin{array}{l} \mathrm{Total effective rate}\\ =\left(\mathrm{Significant improvement}+\mathrm{Effective}\right)/\mathrm{Total cases}\times 100\% \end{array}$ .

##### 2.6.1.2 Anxiety symptoms

Evaluated before treatment and 1 month after treatment using the HAMA (14). The scale includes 14 items, scored on a 0–4 scale, with a total score range of 0–56. Higher scores indicate more severe anxiety. Scores ≤7 are considered no anxiety, 7–14 suggest mild anxiety, 15–21 indicate moderate anxiety, 21–29 indicate severe anxiety, and ≥29 indicate very severe anxiety.

#### 2.6.2 Secondary outcomes

##### 2.6.2.1 Depression symptoms

Assessed using HAMD (17), which includes 17 items, such as early awakening, somatic symptoms, agitation, etc. A total score <7 indicates no depression, 7–17 indicates mild depression, 18–24 indicates moderate depression, and >24 indicates severe depression.

##### 2.6.2.2 Sleep quality

Sleep quality was assessed before treatment and 1 month after treatment using the PSQI (14). The PSQI consists of 7 components and 18 items, each scored from 0 to 3. The total score ranges from 0 to 21, with higher scores indicating poorer sleep quality.

##### 2.6.2.3 PSG parameters

One day before treatment and 1 month after treatment, from 9 PM to 7 AM the next day, PSG monitoring was conducted using the Alice 6LDE multi-channel sleep monitoring system (Philips Respironics). Sleep staging was performed according to the American Academy of Sleep Medicine (AASM) scoring manual (2017 version) (18), with independent scoring by two certified sleep technicians (inter-rater reliability *κ* = 0.85). The parameters measured were TST, awake time (AWT), and SE.

##### 2.6.2.4 Neurotransmitter levels

Blood samples (3 mL) were collected from the patients’ fasting antecubital veins between 7:00 AM and 8:00 AM before treatment and at the same time window (7:00 AM–8:00 AM) 1 month after treatment. After centrifugation (centrifugal radius: 10 cm, speed: 3500 rpm, time: 15 min), the upper serum was collected. The levels of NE, 5-HT, and NPY were measured using enzyme-linked immunosorbent assay kits from Beijing Lyer Biopharmaceutical Technology Co., Ltd. Samples were processed immediately after collection to minimize circadian rhythm-related variability.

#### 2.6.3 Safety outcomes

Adverse events during treatment, including dizziness, pain at the puncture site, and transient upper limb numbness, were recorded and graded according to the Common Terminology Criteria for Adverse Events (CTCAE) (19):

1. Dizziness
  - Grade 1: mild dizziness not interfering with daily activities.
  - Grade 2: moderate dizziness interfering with daily activities but preserving self-care ability (e.g., requiring temporary pauses in work or activities for rest, yet retaining independent mobility and self-care).
  - Grade 3: severe dizziness preventing daily activities and necessitating assistance (e.g., inability to stand or walk independently, partial dependence on assistance for daily living).
  - Grade 4: life-threatening dizziness requiring urgent intervention (e.g., syncope or coma secondary to dizziness).
  - Grade 5: Death related to dizziness.
2. Pain at the puncture site
  - Grade 1: mild pain not interfering with daily activities; no analgesics required.
  - Grade 2: moderate pain interfering with daily activities; non-opioid analgesics (e.g., acetaminophen) required for relief. Pain may limit but not fully prevent routine tasks.
  - Grade 3: significantly impairing daily activities; opioid analgesics required. Pain markedly disrupts normal functioning and sleep.
  - Grade 4: disabling pain or severe complications (e.g., localized muscle spasms, restricted joint mobility) requiring specialized interventions (e.g., surgery).
  - Grade 5: death related to puncture site pain.
3. Transient upper limb numbness
  - Grade 1: occasional mild numbness triggered by specific movements or postures, with no functional impairment.
  - Grade 2: frequent numbness without functional limitation; minimal impact on strength, coordination, or sensation.
  - Grade 3: frequent numbness with sensory or motor dysfunction (e.g., reduced sensitivity to temperature/touch, weakened grip strength, difficulty lifting the arm), impairing daily activities.
  - Grade 4: severe sensory or motor dysfunction (e.g., inability to grasp objects, difficulty writing), significantly compromising quality of life.
  - Grade 5: death related to upper limb numbness.

### 2.7 Statistical analysis

Data were processed using SPSS 26.0 software (IBM Corp.). Normality and homogeneity of variance were assessed using Shapiro–Wilk and Levene’s tests, respectively. For normally distributed data, results are presented as mean ± standard deviation, and between-group comparisons were made using independent *t*-tests or one-way analysis of variance (with *post hoc* Tukey HSD tests for multiple comparisons). Effect sizes were calculated using Cohen’s *d* (thresholds: *d* = 0.2 small, 0.5 medium, 0.8 large). For non-normally distributed data, results are presented as median (quartile range) [M(P25, P75)], and between-group comparisons were made using Mann–Whitney *U* tests or Kruskal-Wallis tests (with Dunn-Bonferroni correction for pairwise comparisons). Categorical data are presented as count (percentage) [n (%)], and between-group comparisons were made using chi-square tests (with Yates’ continuity correction for small cell counts) or Fisher’s exact probability method. Missing data (<5% across all variables) were addressed using last observation carried forward (LOCF). A *p* value < 0.05 (two-tailed) was considered statistically significant. All analyses followed intention-to-treat principles, and 95% confidence intervals (CIs) are reported for primary outcomes.

## 3 Results

### 3.1 Comparison of general data between the two groups

There were no significant differences between the two groups in terms of demographic and clinical characteristics. Variables such as gender, age, disease duration, smoking status, alcohol consumption, body mass index, marital status, occupation, environmental factors, and comorbidities were similar between the research and control groups (all *p* > 0.05; Cohen’s d ranged from 0.02 to 0.20, *η*^2^ < 0.01), indicating negligible effect sizes and confirming baseline comparability. Specifically:

- Gender distribution: the research group had 35 males (54.69%) and 29 females (45.31%), while the control group had 31 males (48.44%) and 33 females (51.56%).
- Age: the average age in the research group was 37.09 ± 9.38 years, while in the control group, it was 36.83 ± 9.07 years. This slight difference was not statistically significant (*p* = 0.874; Cohen’s d = 0.03).
- Disease duration: the research group had an average disease duration of 62.19 ± 12.87 months, while the control group had 63.04 ± 13.25 months. Again, no significant difference (*p* = 0.713; Cohen’s d = −0.06).
- Comorbidities: there were no significant differences in comorbid conditions like hypertension (4 vs. 6 cases in the research and control groups, respectively; *η*^2^ = 0.01) or diabetes (7 vs. 9 cases in the research and control groups, respectively; *η*^2^ = 0.02).

These similarities suggest that the two groups were comparable at baseline, making the subsequent treatment comparisons valid. The sample size of 128 was determined through a power analysis (*α* = 0.05, *β* = 0.01) with a 15% dropout rate adjustment, achieving a statistical power >80% for detecting clinically meaningful differences. The details are shown in Table 1.

**Table 1:** Comparison of general data between the two groups.

Item	Research group (*n* = 64)	Control group (*n* = 64)	t/χ^2^	*p*	Power	Cohen’s d/η^2^	r
Gender (Male) (*n*)	35	31	0.500	0.480	0.780	0.093	0.504
Age (years)	37.09 ± 9.38	36.83 ± 9.07	−0.159	0.874	0.763	0.068	0.074
Disease duration (months)	62.19 ± 12.87	63.04 ± 13.25	0.368	0.713	0.692	−0.065	−0.063
Smoking (*n*)	11	13	0.205	0.651	0.899	8.392	0.063
Alcohol consumption (*n*)	9	12	0.513	0.474	0.907	4.061	0.054
Body Mass Index (kg/m^2^)	23.87 ± 2.37	23.38 ± 2.56	−1.124	0.263	0.709	0.199	0.099
Duration of illness (months)	10.63 ± 2.17	10.54 ± 2.09	−0.239	0.812	0.693	0.542	0.121
Marital status (Married, n)	35	32	0.282	0.595	0.787	50.001	0.100
Employment status (*n*)			0.709	0.702	0.776	9.249	0.220
Farming	20	22					
Freelance	35	36					
Stable employment	9	6					
Environmental factors (*n*)			0.136	0.712	0.745	3.281	0.153
Noisy	40	42					
Quiet	24	22					
Comorbidities (*n*)							
Hypertension	4	6	0.434	0.510	0.937	9.381	0.523
Diabetes	7	9	0.286	0.593	0.920	10.940	0.554

### 3.2 Comparison of clinical efficacy between the two groups

At 1 month after treatment, the research group showed a significantly higher total effective rate than the control group, indicating better overall treatment outcomes.

- Significant effect: the research group achieved a 57.81% significant effect rate (37 out of 64 patients), while the control group had 29.69% (19 out of 64 patients).
- Effective: the research group had 40.63% (26 patients) classified as effective, compared to 59.38% (38 patients) in the control group.
- Ineffective: the research group had only 1 patient (1.56%) with no improvement, while 7 patients (10.94%) in the control group were classified as ineffective.

Overall, the total effective rate for the research group was 98.44%, significantly higher than the 89.06% total effective rate in the control group (*p* = 0.028), confirming that the treatment was more effective in the research group, as shown in Table 2.

**Table 2:** Comparison of clinical efficacy between the two groups ( $\bar{x}$ ±s, points).

Item	Research Group (*n* = 64)	Control Group (*n* = 64)	*χ* ^2^	*p*	Power	Cohen’s d	*r*
Significant effect	37 (57.81%)	19 (29.69%)					
Effective	26 (40.63%)	38 (59.38%)					
Ineffective	1 (1.56%)	7 (10.94%)					
Total effective rate	63 (98.44%)	57 (89.06%)	4.800	0.028	0.628	2.123	0.009

### 3.3 Comparison of anxiety and depression between the two groups

Before treatment: Anxiety and depression levels were comparable between the two groups, as reflected by the similar scores on the anxiety and depression scales (*p* > 0.05).

- Anxiety: Both groups had a baseline anxiety score around 40 (Research Group: 40.23 ± 12.09, Control Group: 40.17 ± 12.52), showing no significant difference (*p* = 0.978).
- Depression: Similarly, baseline depression scores were also close (Research Group: 19.34 ± 2.13, Control Group: 19.28 ± 2.76), with no significant difference (*p* = 0.891).

One month after treatment: Significant reductions in both anxiety and depression were observed in the research group compared to the control group.

- Anxiety: the research group showed a significant decrease in anxiety (9.36 ± 2.34) compared to the control group (11.87 ± 2.71) (*p* < 0.001).
- Depression: similarly, the research group experienced a substantial reduction in depression (6.87 ± 2.01) compared to the control group (8.09 ± 2.04) (*p* < 0.001).

**Figure 1:**
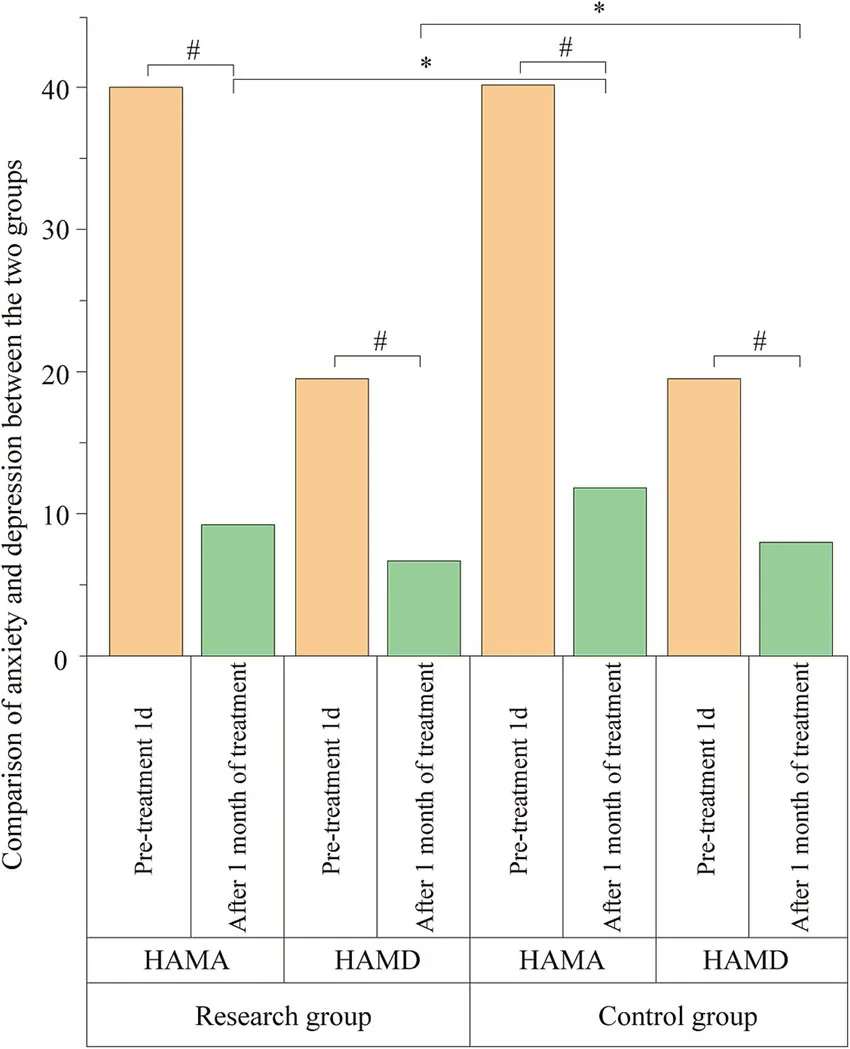
Comparison of anxiety and depression between the two groups. ^#^*p* < 0.05 compared to the same group before treatment (1 day); *p < 0.05 compared to the control group at 1 month after treatment.

This indicates that the intervention had a more significant effect in reducing anxiety and depression in the research group. Results were shown in Table 3 and Figure 1.

**Table 3:** Comparison of anxiety and depression between the two groups ( $\bar{x}$ ±s, points).

Item	Time	Research group (*n* = 64)	Control group (*n* = 64)	*t*	*p*	Power	Cohen’s d/η^2^	*r*
Anxiety	Before treatment (1 day)	40.23 ± 12.09	40.17 ± 12.52	−0.028	0.978	0.761	0.705	0.102
	1 month after treatment	9.36 ± 2.34	11.87 ± 2.71	5.608	<0.001	0.639	−0.991	−0.034
Depression	Before treatment (1 day)	19.34 ± 2.13	19.28 ± 2.76	−0.138	0.891	0.786	−0.602	−0.288
	1 month after treatment	6.87 ± 2.01	8.09 ± 2.04	3.408	<0.001	0.693	0.024	0.012

### 3.4 Comparison of sleep quality between the two groups

Before treatment: there were no significant differences in sleep quality between the two groups at baseline, as indicated by their similar PSQI scores (*p* > 0.05).

- Research Group: 17.92 ± 2.01
- Control Group: 17.86 ± 1.97

One month after treatment: the research group showed a marked improvement in sleep quality, with a significant reduction in PSQI scores (5.74 ± 1.64) compared to the control group (8.03 ± 1.86) (*p* < 0.001).

**Figure 2:**
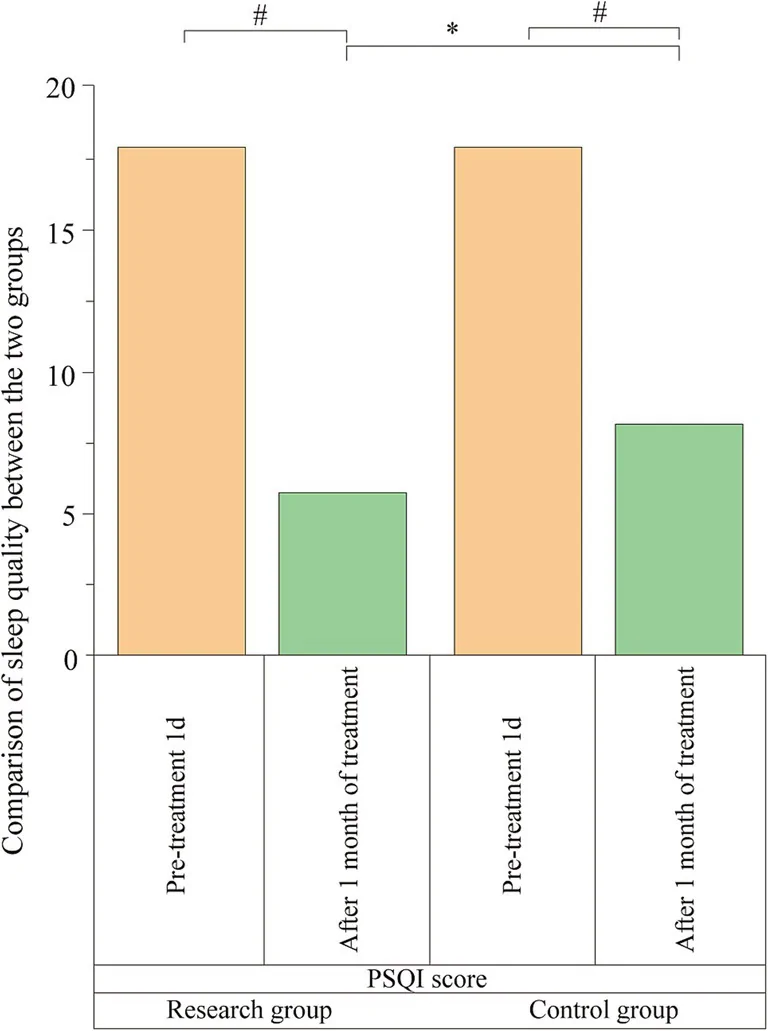
Comparison of sleep quality between the two groups. ^#^*p* < 0.05 compared to the same group before treatment (1 day); *p < 0.05 compared to the control group at 1 month after treatment.

This suggests that the research group experienced a significantly greater improvement in sleep quality after treatment, as evidenced by the lower PSQI scores. Results were shown in Table 4 and Figure 2.

**Table 4:** Comparison of sleep quality between the two groups ( $\bar{x}$ ±s, points).

Item	Time	Research group (*n* = 64)	Control group (*n* = 64)	*t*	*p*	Power	Cohen’s d	*r*
Sleep Quality	Before treatment (1 day)	17.92 ± 2.01	17.86 ± 1.97	−0.171	0.865	0.663	−1.306	−0.547
	1 month after treatment	5.74 ± 1.64	8.03 ± 1.86	7.388	<0.001	0.770	0.030	0.015

### 3.5 Comparison of sleep parameters between the two groups

Before treatment: there were no significant differences in the sleep parameters of TST, AWT, and SE between the two groups.

- TST: the research group had 327.04 ± 30.91 min and the control group had 324.28 ± 31.19 min of TST (*p* = 0.616).
- AWT: the research group spent 66.83 ± 16.86 min awake, while the control group spent 65.91 ± 15.94 min awake (*p* = 0.752).
- SE: SE was slightly higher in the control group (69.64 ± 11.28%) compared to the research group (68.45 ± 10.04%), but the difference was not statistically significant (*p* = 0.530).

One month after treatment: there were significant improvements in all sleep parameters in the research group:

- TST: The research group had a significantly longer TST (429.76 ± 33.22 min) compared to the control group (391.13 ± 30.76 min) (*p* < 0.001).
- AWT: The research group experienced significantly less wake time (19.87 ± 5.61 min) compared to the control group (24.34 ± 5.23 min) (*p* < 0.001).
- SE: SE was higher in the research group (90.23 ± 13.29%) compared to the control group (86.34 ± 12.84%) (*p* < 0.001).

These findings suggest that the research group experienced a much more significant improvement in sleep duration, efficiency, and reduced wake time. Results were shown in Table 5.

**Table 5:** Comparison of sleep parameters between the two groups ( $\bar{x}$ ±s).

Item	Parameter	Research group (*n* = 64)	Control group (*n* = 64)	*t*	*p*	Power	Cohen’s d	*r*
TST (min)	Before treatment (1 day)	327.04 ± 30.91	324.28 ± 31.19	−0.503	0.616	0.663	1.207	0.517
	1 month after treatment	429.76 ± 33.22	391.13 ± 30.76	−6.826	<0.001	0.712	0.089	0.044
AWT (min)	Before treatment (1 day)	66.83 ± 16.86	65.91 ± 15.94	−0.317	0.752	0.743	−0.824	−0.381
	1 month after treatment	19.87 ± 5.61	24.34 ± 5.23	4.662	<0.001	0.659	0.056	0.028
SE (%)	Before treatment (1 day)	68.45 ± 10.04	69.64 ± 11.28	0.630	0.530	0.671	0.731	0.343
	1 month after treatment	90.23 ± 13.29	86.34 ± 12.84	−1.684	<0.001	0.631	0.298	0.27

### 3.6 Comparison of neurotransmitter levels between the two groups

Before treatment: there were no significant differences in the levels of neurotransmitters, including NE, 5-HT, and NPY, between the two groups (*p* > 0.05).

- NE: Research group: 235.98 ± 48.63 pg./mL, Control group: 237.91 ± 51.21 pg./mL (*p* = 0.891).
- 5-HT: Research group: 108.43 ± 13.23 ng/mL, Control group: 109.56 ± 14.02 ng/mL (*p* = 0.682).
- NPY: Research group: 345.21 ± 62.44 pg./mL, Control group: 348.12 ± 64.21 pg./mL (*p* = 0.872).

One month after treatment: significant changes in neurotransmitter levels were observed in the research group compared to the control group:

- NE: The research group showed a significant increase in NE levels (289.43 ± 51.68 pg./mL) compared to the control group (253.78 ± 57.12 pg./mL) (*p* < 0.05).
- 5-HT: The research group exhibited significantly higher 5-HT levels (138.56 ± 19.73 ng/mL) compared to the control group (124.93 ± 18.44 ng/mL) (*p* < 0.05).
- NPY: The research group had higher NPY levels (453.21 ± 73.41 pg./mL) compared to the control group (402.34 ± 68.12 pg./mL) (*p* < 0.05).

These changes in neurotransmitter levels suggest that the treatment in the research group had a positive effect on the balance of neurotransmitters associated with mood and sleep regulation. Results were shown in Table 6.

**Table 6:** Comparison of neurotransmitters between the two groups ( $\bar{x}$ ±s).

Item	Group	Research group (*n* = 64)	Control group (*n* = 64)	*t*	*p*	Power	Cohen’s d	*r*
NE (pg/mL)	Before Treatment (1 day)	543.28 ± 31.73	541.83 ± 35.98	−0.242	0.809	0.718	−1.044	−0.463
	1 Month After Treatment	356.32 ± 30.64	391.28 ± 36.09	5.908	< 0.001	0.709	0.043	0.021
5-HT (pg/mL)	Before Treatment (1 day)	30.39 ± 9.47	31.73 ± 9.03	0.828	0.409	0.707	1.123	0.490
	1 Month After Treatment	119.67 ± 9.65	108.37 ± 10.46	−6.352	< 0.001	0.641	−0.045	−0.022
NPY (pg/mL)	Before Treatment (1 day)	119.32 ± 24.28	121.34 ± 27.09	0.444	0.658	0.701	0.400	0.196
	1 Month After Treatment	158.93 ± 22.09	149.86 ± 23.38	−2.256	0.026	0.771	−0.079	−0.039

### 3.7 Comparison of adverse reactions between the two groups

During the treatment period, the research group reported the following adverse reactions: 2 cases of mild dizziness, 1 case of pain at the puncture site, and 1 case of transient upper limb numbness. The overall incidence of adverse reactions in the research group was 6.25% (4/64). In the control group, there were 2 cases of mild dizziness, and the incidence of adverse reactions was 3.13% (2/64). There were no significant differences in the incidence of adverse reactions between the two groups (*χ*^2^ = 0.699, *p* = 0.403, Power = 0.920, Cohen’s d = 5.492, *r* = 0.094).

## 4 Discussion

In the case of GAD with comorbid sleep disorders, the sympathetic nervous system remains in a highly active state for an extended period, which can easily trigger anxiety-related sleep disturbances, light sleep, and frequent nocturnal awakenings. These issues can adversely affect the physical and mental health of patients, such as causing attention deficits, memory decline, and the emergence of negative emotions, which indirectly exacerbate sleep disorders (20). Although traditional drug treatments (such as anxiolytics and sedatives) are effective in the short term, long-term use often leads to drug dependence, tolerance, and other side effects.

Eszopiclone, a short-acting benzodiazepine drug, has sedative, hypnotic, and anxiolytic effects, with a sedative-hypnotic effect 2.4–4 times stronger than that of nitrazepam. Its drawback is the potential for drug dependence; long-term use of eszopiclone can lead to psychological and physical dependence (21). Benzodiazepines primarily act on *γ*-aminobutyric acid (GABA) receptors in the brain, enhancing GABA-mediated inhibitory neurotransmission to reduce neuronal excitability, thereby exerting anxiolytic, sedative, and hypnotic effects. These drugs effectively shorten sleep latency, reduce nighttime awakenings, increase TST, and improve sleep quality while alleviating anxiety. However, prolonged use is associated with tolerance, dependence, and withdrawal symptoms (e.g., insomnia, anxiety, agitation) upon discontinuation. Adverse effects such as daytime drowsiness, dizziness, fatigue, and impaired cognitive or psychomotor function may also occur (22). A study by Zou et al. (23) demonstrated that diazepam outperformed alprazolam in improving postoperative sleep disturbances in glioma patients, potentially linked to reduced expression of 5-HT and NE. Xiao et al. (24) reported that lorazepam combined with risperidone significantly improved psychiatric symptoms, sleep quality, mental status, and psychological well-being in schizophrenia patients with comorbid sleep disorders. Non-Benzodiazepine Hypnotics selectively target specific GABA receptor subunits, differing from benzodiazepines in their binding sites. They exhibit potent sedative-hypnotic effects with minimal disruption to normal sleep architecture. These agents rapidly induce sleep, enhance SE, and improve sleep quality, particularly for anxiety-related sleep initiation or maintenance difficulties, while posing lower risks of respiratory depression during sleep. Common side effects include bitter taste, dry mouth, and headache. Chronic use may still carry dependency risks, and abrupt discontinuation can trigger rebound insomnia (25). Gu et al. (26) found that escitalopram combined with zopiclone significantly alleviated anxiety symptoms, improved sleep quality, and enhanced quality of life in anxiety disorder patients with insomnia, albeit with associated adverse effects. Wang et al. (27) reported satisfactory outcomes with zolpidem-trazodone combination therapy in insomnia patients, reducing sleep disturbance severity and improving sleep quality, though adverse effects were noted. Therefore, it is important to actively adopt effective methods for treating GAD with comorbid sleep disturbances, which holds significant clinical value.

SGB, an emerging neuroregulation technique, involves blocking the stellate ganglion with local anesthetics to effectively suppress the overactivity of the sympathetic nervous system, thus improving sleep quality (28). SGB not only reduces the patient’s dependence on medications but may also indirectly affect emotional regulation areas in the brain through the modulation of the central nervous system, providing a new perspective for treating anxiety-related sleep disorders (28).

One recent study indicated that SGB combined with CBT significantly improved treatment outcomes in patients with persistent insomnia, also enhancing sleep quality (29). A study by Yu Gu et al. (30) found that ultrasound-guided SGB effectively improved the clinical symptoms of elderly patients with insomnia and significantly increased sleep quality.

The results of this study show that 1 month after treatment, the total effective rate of the research group was higher than that of the control group (*p* < 0.05), suggesting that SGB can effectively improve the clinical symptoms of patients with GAD and comorbid sleep disturbances, with significant therapeutic effects.

This finding may be related to the following factors: the stellate ganglion is composed of the inferior cervical ganglion, middle cervical ganglion, and the first and second thoracic ganglia. In practice, a C6-level block involves the middle cervical ganglion or sympathetic trunk. The local anesthetic can diffuse to the stellate ganglion, and its post-ganglionic fibers are widely distributed in the skin tissues from the 3rd cervical vertebra to the 12th thoracic vertebra. SGB involves injecting lidocaine into the connective tissue innervated by the stellate ganglion, which blocks sympathetic nerves in the chest, upper limbs, face, and head and neck, thus achieving the therapeutic purpose for GAD with comorbid sleep disturbances (30).

Liu et al. (31) found that SGB effectively improved sleep quality in patients with sleep disorders. Gu et al. (30) reported that ultrasound-guided SGB could improve the objective and subjective sleep quality of elderly patients after thoracoscopic surgery for lung cancer, alleviate stress responses and sleep disturbances, and promote recovery.

The results of this study show that 1 month after treatment, the research group exhibited significantly lower anxiety and depression levels than the control group (*p* < 0.05), suggesting that SGB can effectively improve anxiety and depression in patients with GAD and comorbid sleep disturbances. The PSQI is a widely used subjective assessment tool that evaluates sleep based on factors such as difficulty falling asleep, sleep duration, self-assessment of sleep quality, and the use of sleep medications. It has high reliability in assessing sleep quality and screening sleep disorders in clinical practice and correlates well with the gold standard of PSG. Yan et al. (32) reported that SGB improved sleep disturbances in patients after radical surgery for gastrointestinal malignancies. Yang et al. (33) noted that preoperative ultrasound-guided SGB could improve postoperative sleep quality and analgesia in breast cancer patients.

The results of this study show that 1 month after treatment, the PSQI score of the research group was significantly lower than that of the control group (*p* < 0.05), indicating that SGB can effectively improve sleep quality in patients with GAD and comorbid sleep disturbances.

PSG is the gold standard for sleep monitoring, capturing brain activity and respiratory parameters, and generating an electroencephalogram (EEG) to classify sleep stages and reflect sleep quality (34). Rahimzadeh et al. (34) found that SGB was as effective as paroxetine in controlling hot flashes and sleep disturbances in breast cancer survivors, with fewer complications. Liu et al. (31) showed that combining SGB with repetitive transcranial magnetic stimulation for treating post-stroke insomnia patients could reduce PSQI scores, restore polysomnographic indices, and improve patients’ sleep quality and conditions.

In this study, 1 month after treatment, the research group had significantly higher TST and SE and lower AWT compared to the control group (*p* < 0.05), suggesting that SGB can effectively improve the sleep conditions of patients with GAD and comorbid sleep disturbances. The reasons for this may be as follows:

Neurophysiologically, the hypothalamus and thalamus play an important role in regulating the regularity of the biological clock. SGB involves injecting lidocaine into the loose connective tissue at the neck and stellate ganglion, inducing reversible block of the pre-and post-ganglionic fibers and the sympathetic nerves they innervate (31). SGB, under real-time guidance, ensures accurate needle depth, needle position, and needle approach, avoiding damage to important surrounding tissues, nerves, or blood vessels during puncture. Therefore, SGB is a widely applicable, effective, and safe clinical treatment modality. It improves sleep quality in patients with sleep disorders more effectively than conventional drug treatments, is well accepted by patients, and significantly enhances their sleep quality (31).

When there is a disorder in neurotransmitter secretion, it can affect emotional state, hinder the recovery of neurological function, and stimulate a large release of pro-inflammatory factors (35). Studies have shown that changes in the sleep–wake cycle can affect the secretion of various neurotransmitters in the cerebral cortex and brainstem, further impacting sleep quality. A decrease in neurotransmitters such as 5-HT and NPY is a prominent manifestation (35). The results of this study show that 1 month after treatment, the levels of NE in the research group were lower than those in the control group, while the levels of 5-HT and NPY were higher in the research group compared to the control group (*p* < 0.05). This indicates that SGB can effectively regulate NE, 5-HT, and NPY levels in patients with GAD and comorbid sleep disturbances, restoring neurotransmitter levels. This is one of the mechanisms by which SGB treats GAD with comorbid sleep disturbances.

The reason for this may be that acetylcholine, 5-hydroxytryptamine (5-HT), and GABA are all inhibitory neurotransmitters, widely distributed in the pineal gland and hypothalamus, and play important roles in initiating and maintaining sleep. U-SGB can suppress sympathetic fibers by blocking the pre-ganglionic fibers of sympathetic ganglia that innervate areas such as the head and neck. Furthermore, the use of ultrasound guidance can improve the success rate of the block, effectively regulating the connections between the brain cortex, brainstem reticular ascending inhibitory system, hypothalamus, and cortical projection pathways. This helps prevent depolarization of hypothalamic-cortical projection neurons, providing a better environment for neurotransmitter secretion in the cerebral cortex and brainstem centers of elderly patients with insomnia, thus inhibiting the excitability of the sleep center, promoting the prolongation of sleep time, and improving sleep quality.

U-SGB suppresses sympathetic hyperactivity by selectively blocking preganglionic fibers innervating the head and neck regions, with ultrasound guidance significantly enhancing procedural precision and success rates. This intervention modulates interactions among the cerebral cortex, brainstem ascending reticular inhibitory system, and hypothalamic-cortical pathways, preventing hyperpolarization of hypothalamic-cortical projection neurons and creating an optimal microenvironment for neurotransmitter secretion in cortical and brainstem nuclei of elderly insomnia patients. By inhibiting excitability of sleep-regulatory centers, SGB prolongs total sleep duration and improves sleep quality (36). Mechanistically, SGB directly suppresses excessive sympathetic activity at the stellate ganglion, reducing NE release and dampening stress responses to induce systemic relaxation. Simultaneously, it regulates the hypothalamic–pituitary–adrenal (HPA) axis, modulating secretion of corticotropin-releasing hormone (CRH), adrenocorticotropic hormone (ACTH), and cortisol—hormones intricately linked to the metabolism and balance of neurotransmitters such as dopamine and GABA. Additionally, SGB dilates cephalic, cervical, and upper limb vasculature, improving regional perfusion to enhance oxygen and nutrient delivery while facilitating metabolic waste clearance. These hemodynamic effects stabilize neuronal function and support neurotransmitter synthesis, release, and metabolism, collectively contributing to symptom alleviation through both direct and indirect neuromodulatory pathways (31).

This study further found that there were no significant differences in adverse reactions between the two groups (*p* > 0.05), suggesting that SGB treatment for GAD with comorbid sleep disturbances does not increase the occurrence of adverse effects.

However, this study has several limitations. First, the sample size was relatively small, and the single-center design may restrict the generalizability of the findings. Importantly, the strict exclusion of psychiatric comorbidities (e.g., depression, PTSD, and other anxiety disorders) and physical illnesses resulted in a homogeneous study population; while this enhanced internal validity, it limits the applicability of our conclusions to real-world patients with complex psychiatric or medical conditions. Additionally, the short study duration precludes conclusions about long-term efficacy, and the mechanisms underlying SGB in treating GAD with sleep disturbances (e.g., neuroinflammatory or epigenetic pathways) remain unexplored. Therefore, future multicenter studies with larger, more diverse cohorts (including patients with comorbid psychiatric disorders), extended follow-up periods, and mechanistic investigations are warranted to validate and expand upon these results.

In conclusion, SGB effectively alleviates clinical symptoms of GAD with comorbid sleep disturbances in patients without concurrent depression. SGB significantly reduces anxiety severity, improves sleep quality (as evidenced by reduced PSQI scores and enhanced polysomnographic parameters), and modulates neurotransmitter levels (decreased NE, elevated 5-HT and NPY), suggesting a potential mechanism involving sympathetic nervous system regulation. The exclusion of psychiatric comorbidities, particularly depression, strengthens the specificity of these findings to GAD with sleep disruption.

## Data Availability

The original contributions presented in the study are included in the article/Supplementary material, further inquiries can be directed to the corresponding author.

## Supplementary material

SUPPLEMENTARY FIGURE 1 The study flow diagram.
