# Correction: Efficacy and exploratory analysis of potential mechanisms of stellate ganglion block in alleviating sleep disturbance in generalized anxiety disorder: a randomized controlled trial excluding comorbid depression

**DOI:** 10.3389/fneur.2026.2015094

**Published:** 2026-10-12

**Authors:** Na Liu, Qinying Ma, Moqing Zhou, Lin Yang, Wenyuan Wang, Yanyong Wang

**Affiliations:** Department of Neurology, The First Hospital of Hebei Medical University, Shijiazhuang, Hebei, China

**Keywords:** stellate ganglion block, generalized anxiety disorder, sleep disturbance, efficacy, mechanism

In the published article, the **Funding** statement was incorrectly published as:

The author(s) declare that financial support was received for the research and/or publication of this article. This study was supported by Hebei Province Natural Science Foundation biological medicine joint fund cultivation project (H2021206325).

The correct statement reads:

The author(s) declared that financial support was received for this work and/or its publication. This study was supported by the S&T Program of Hebei: Central Government-Guided Local Science and Technology Development Fund (Free Exploration Basic Research), No. 236Z7733G, and the 2024 Government-Funded Training Program for Outstanding Talents in Clinical Medicine, No. ZF2024140.

The original version of this article has been updated.

