# Supplementary figures and images for: Efficacy and exploratory analysis of potential mechanisms of stellate ganglion block in alleviating sleep disturbance in generalized anxiety disorder: a randomized controlled trial excluding comorbid depression
Source: Front Neurol. 2025 May 7;16:1554841. doi: 10.3389/fneur.2025.1554841 (PMC12092344; doi:10.3389/fneur.2025.1554841)

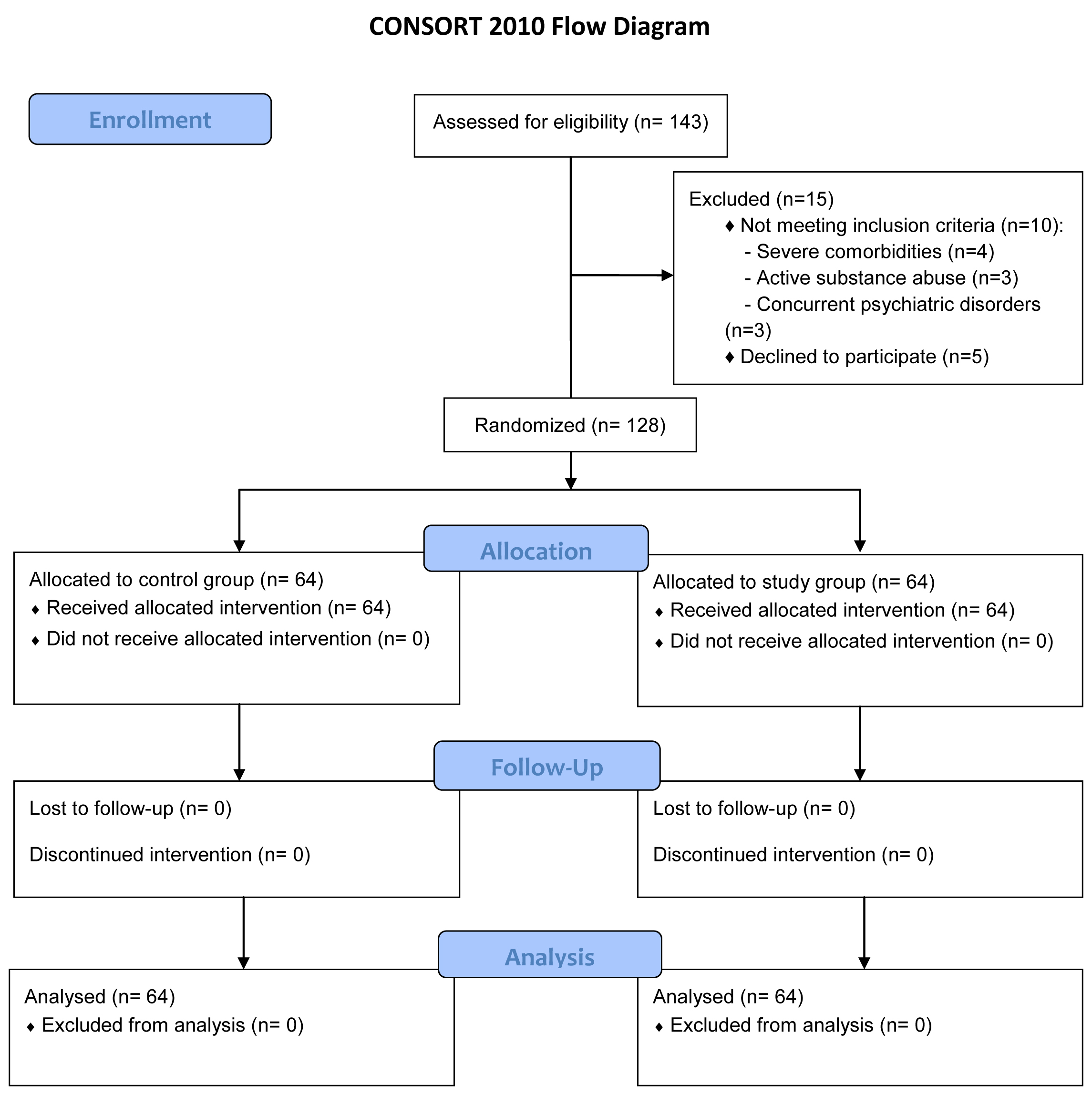

Supplement: SUPPLEMENTARY FIGURE 1 — The study flow diagram. [file Image_1.JPEG]
